# Guitar: An R/Bioconductor Package for Gene Annotation Guided Transcriptomic Analysis of RNA-Related Genomic Features

**DOI:** 10.1155/2016/8367534

**Published:** 2016-04-28

**Authors:** Xiaodong Cui, Zhen Wei, Lin Zhang, Hui Liu, Lei Sun, Shao-Wu Zhang, Yufei Huang, Jia Meng

**Affiliations:** ^1^Department of Electrical and Computer Engineering, University of Texas at San Antonio, San Antonio, TX 78230, USA; ^2^Department of Biological Sciences, HRINU, SUERI, Xi'an Jiaotong-Liverpool University, Suzhou 215123, China; ^3^Institute of Integrative Biology, University of Liverpool, Liverpool L69 3BX, UK; ^4^School of Information and Electrical Engineering, China University of Mining and Technology, Xuzhou 221116, China; ^5^School of Information Engineering, Yangzhou University, Yangzhou, Jiangsu 225127, China; ^6^School of Automation, Northwestern Polytechnical University, Xi'an, Shaanxi 710027, China

## Abstract

Biological features, such as genes and transcription factor binding sites, are often denoted with genome-based coordinates as the genomic features. While genome-based representation is usually very effective in correlating various biological features, it can be tedious to examine the relationship between RNA-related genomic features and the landmarks of RNA transcripts with existing tools due to the difficulty in the conversion between genome-based coordinates and RNA-based coordinates. We developed here an open source* Guitar* R/Bioconductor package for sketching the transcriptomic view of RNA-related biological features represented by genome based coordinates. Internally,* Guitar* package extracts the standardized RNA coordinates with respect to the landmarks of RNA transcripts, with which hundreds of millions of RNA-related genomic features can then be efficiently analyzed within minutes. We demonstrated the usage of* Guitar* package in analyzing posttranscriptional RNA modifications (5-methylcytosine and N6-methyladenosine) derived from high-throughput sequencing approaches (MeRIP-Seq and RNA BS-Seq) and show that RNA 5-methylcytosine (m^5^C) is enriched in 5′UTR. The newly developed* Guitar* R/Bioconductor package achieves stable performance on the data tested and revealed novel biological insights. It will effectively facilitate the analysis of RNA methylation data and other RNA-related biological features in the future.

## 1. Introduction

Genome-based coordinates, which consist of the name of chromosome and the starting/ending coordinates, have been widely used to denote the genomic location of various biological features, such as genes, SNPs, and transcription factor binding sites (TFBS). With genome-based coordinates, the relationship between different biological features can be easily inferred. Currently, genomic features (biological features represented by genome-based coordinates) have become the basis of many bioinformatics tools in various biological data processing pipelines, and dedicated types of operation are also available [1]. While genome-based coordinates are very useful for analysis of genome related biological features, it can still be tedious for analysis or visualization of RNA-related features, such as RNA N6-methyladenosine (m^6^A) and RNA 5-methylcytosine (m^5^C) [2].

As an emerging layer of gene expression regulation, posttranscriptional RNA modifications, including m^6^A and m^5^C, are recently found to play various important roles in a number of biological processes, such as translation efficiency [3], microRNA processing [4], RNA-protein interaction [5], RNA stability [6], and pluripotency [7]. Together with the development of new sequencing approaches [8–11] for unbiased profiling of the posttranscriptional RNA modifications, a number of bioinformatics tools [12, 13] have been created for interpretation of these datasets. A mammalian RNA methylation database [14] has been created that paved the way for a systematic understanding of the RNA methylome regulation mechanism [15]; however, to our knowledge, no bioinformatics effort has been made specifically for effective visualization of RNA methylation features from global level. Conceivably, the functions of RNA-related features are likely to be related to the landmarks of RNA transcripts, that is, transcription starting site (TSS), start codon, stop codon, and transcription ending site (TES), and the existing tools developed for genome-based features are not effective for analysis of RNA methylation data.

Compared with genome-regulated biological features (e.g., histone modifications and TFBS), visualization of RNA-related features (such as RNA methylation sites) represented in genomic coordinates is nontrivial due to the following reasons:

- *Increased Ambiguity due to Isoforms*. The ambiguity in genome-based coordinates is often due to the repeats. Compared with genome, transcriptome has increased complexity due to transcript isoform and alternative splicing. It is possible that an RNA methylation site falls into the CDS of one transcript but on the 3′UTR of another isoform transcript. It is known that many genes have very large number of isoform transcripts, so it may be impossible to specifically assign a genomic feature aligned on this gene to a particular isoform transcript.
- *Variation in Transcript Length*. In human and mouse, transcripts vary greatly in size. It is important to notice that 1000 base pairs (bp) from start codon may be a long distance for shorter genes but not for the longer ones. For genomic coordinates, bp has been shown to be a reasonable unit; however, when measuring distance between two RNA-related features, a relative unit standardized by the entire width of the transcript may be more suitable. This idea has been used widely today in many studies. Please note that the technical resolution remains the same when measured in bp on shorter or longer transcripts. When a standardized coordinate is used, longer transcripts actually obtain a higher resolution in terms of standardized coordinate compared with the shorter transcripts. Compared with a transcript with 20 k bp, it is more difficult to tell whether the RNA methylation site on a 200 bp transcript is close to its stop codon, even though the technical resolution remains the same.
- *Complexity in Landmarks of RNA Transcript*. For histone modifications and transcription factor binding sites, landmarks of interests are often the transcription starting site (TSS) and transcription ending sites (TES). For RNA methylation, two additional landmarks are the start codon and stop codon of mRNA and the setting is further complicated by the existence of long noncoding RNA (lncRNA) and various small RNA families. Furthermore, some mRNAs may not have 3′UTR or 5′UTR. Additionally, the 5′UTR, CDS, and 3′UTR are apparently of different length for most genes, so the 3 components need to be standardized independently when summarized or compared with other transcripts. There are already tools, such as ngs.plot [16], developed to handle TSS and TES, but to our knowledge, none supports more detailed structure (start codon and stop codon). It is important to include all RNA landmarks and discriminate coding and noncoding RNAs when analyzing RNA-related features due to their intrinsic property.

For the aforementioned reasons, a dedicated approach needs to be developed for visualization of RNA methylation data and other RNA-related biological features.

## 2. Design and Implementation

We develop here an open source R package* Guitar* for gene annotation guided transcriptomic analysis of RNA-related genomic features, such as RNA methylation sites denoted in genome-based coordinates. The approach is detailed next.

### 2.1. Guitar Coordinates

**Figure 1:**
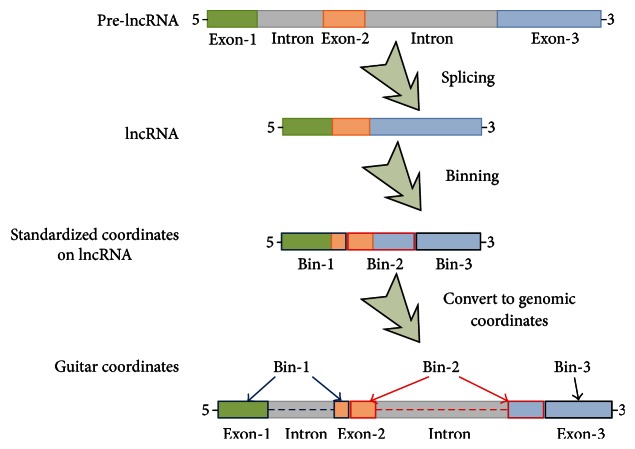
Guitar coordinates. This figure illustrates how the* Guitar* coordinates are generated based on 3 bins on lncRNA transcript. The bins may be split into multiple sections of the transcript represented by* GRangesList* object, which can be conveniently compared with other genome-based features to reflect their distribution. In practice, if 70% of a feature overlap 5′UTR and 30% CDS, then it is likely that, the feature overlaps with more Guitar Coordinates (GCs) composed from 5′UTR than from CDS. As a result, more GCs from 5′UTR will report the overlapping of this feature, and the resulting weight will precisely reflect the 70% and 30% division.

To visualize the multiple RNA-related features together, transcripts of different length need to be standardized in the first place. For this purpose, we constructed the* Guitar* coordinates, which is essentially the genomic projection of the standardized transcriptomic coordinates. Specifically, each component of a single transcript is divided into a number of bins of equal width. For long noncoding RNA, the whole transcript is a single component; for mRNA, there are 3 components, that is, 5′UTR, CDS, and 3′UTR. Their genomic projected coordinates are then obtained with the help of* GenomicFeatures* R/Bioconductor package [1]. Please note that of interest are the mature mRNA and lncRNA, and it is possible that a specific bin may span introns. The generated* Guitar* coordinates are essentially still genome-based coordinates but clearly associated with landmarks of transcript, for example, 0.2 standardized lncRNA length from the TSS. The procedures for generating* Guitar* coordinates are illustrated in Figure 1.

### 2.2. Guitar Coordinates of a Transcriptome

As mentioned previously, for mRNA, of interest are usually 3 components rather than a single one, that is, 5′UTR, CDS, and 3′UTR. Consistently, the* Guitar* coordinates need to be generated separately for all the 3 regions. In order to make the 3 components comparable, each component is standardized independently and contributes to 1/3 of the entire coding transcript (the difference between 5′UTR, CDS, and 3′UTR in size can also be reflected in the analysis by* Guitar* package). For lncRNA, this is not needed and the* Guitar* coordinates are generated for the entire lncRNA.

Due to the existence of isoform ambiguity, the same genomic location may be associated with multiple transcripts and thus related to multiple* Guitar* coordinates. To ensure the specificity of the generated* Guitar* coordinates, filtering of highly ambiguous transcripts may be needed. Two filters are implemented. Firstly, a length filter is implemented to select transcripts longer than a user-defined threshold. This is to ensure the generated* Guitar* coordinates have sufficient resolution from the technology perspective with the data analyzed. For techniques with single-base resolution, this cutoff can be smaller (e.g., 10 bp), while, for techniques with lower resolution, it should be larger (e.g., 100 bp). Secondly, an ambiguity filter is implemented to discard genes with too many isoforms, which can cause ambiguity in the feature assignment stage. The implementation of this filter is mainly to reduce memory usage and save computation time (see Supplementary Material Figure S1 and Table S1 for a comparison of different settings in Supplementary Material available online at http://dx.doi.org/10.1155/2016/8367534). In addition, the filter may be necessary for less-studied species with problematic gene annotation. The second filter can filter out a number of genes with higher isoform ambiguity, and doing so will significantly decrease the memory usage for the constructed* Guitar* coordinates and save computation time. For this purpose, we implemented a simple strategy by counting the number of overlapping transcripts on the genome, that is, checking the number of transcripts sharing exons. In the default setting of* Guitar* package, we filtered out transcripts that overlap with more than 3 other transcripts on the genome. The parameter provides reasonable good results in the data tested and can be easily customized by the user. After applying the ambiguity filter, if a genomic feature still overlaps with multiple transcripts, the ambiguity will be factored in the analysis. Specifically, if a site is located on both CDS and 3′UTR of the same transcript, then the Guitar coordinates associated with those components should have overlapped with that genomic feature, and those coordinates are labeled as associated with the genomic feature with weight 1, indicating the feature is fully associated with this transcript. However, if a genomic feature is associated with *n* transcripts, that is, it overlaps with *n* transcripts, then the overlapping coordinates are associated with that genomic feature with weight 1/*n*, reflecting the ambiguity in association.

### 2.3. Transcriptomic View of Genomic Features

**Figure 2:**
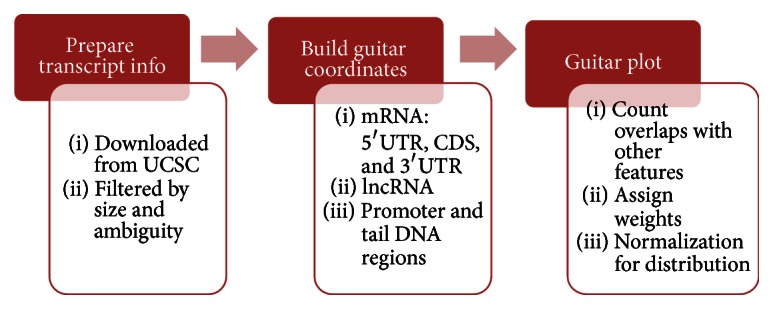
Workflow of* Guitar* package. The gene annotation can be automatically downloaded from Internet or provided as a* TxDb* object [1] by the user. After filtering transcripts that may not provide useful information,* Guitar* coordinates of different components are built independently, with which the actual transcriptomic distribution of genomic features can be observed.

To sketch the transcriptomic view of genomic features, the* Guitar* coordinates can be compared with other genomic features, and the number of overlapped features can then be counted and standardized on mRNA and lncRNA, respectively. The distribution of genomic features on RNA will then be summarized and visualized by* ggplot2* package for quality graphics [17]. We also provided a convenient function GuitarPlot for fast visualization of various genomic features in different formats. The general working procedures for* Guitar* package are shown in Figure 2.

Besides the aforementioned structure, there are a few more useful features provided by* Guitar* package:

- *Including neighborhood DNA regions for comparison*. The neighborhood DNA regions (promoter region and its complementary DNA at the 3′ end) can be optionally included in analysis of* Guitar* package. This will be useful for analyzing genomic features that are related to both DNA and RNA, such as H3K4me3 ChIP-Seq data.
- *Ambiguous Assignment of the Ambiguous Genomic Features*. Due to ambiguity of heterogeneous transcriptome, some genomic features overlap with more than one transcript and thus with ambiguous belongings. In the* Guitar* package, ambiguous features are also counted in the analysis, with their weights equally divided between all the overlapping transcripts. However, we can optionally filter out genomic features overlapping with more than a predefined number of transcripts (Default 5) to completely discard the impact of these highly ambiguous features.
- *Resizing the Components in Visualization*. In practice, different components of mRNA, that is, 5′UTR, CDS, and 3′UTR, are of quite different width. 5′UTR is usually much shorter than 3′UTR and CDS in mouse and human. Although these components are treated independently when building the* Guitar* coordinates, we still calculated the average length of each component across different genes, so the true relative width can be optionally reflected in the plot generated from* Guitar* package; however, doing so may make the 5′UTR region too small for clear observation.
- *Connection with ggplot2 for More Complex Graphics*. The* Guitar* may optionally return intermediate results, which can be reused in the ggplot2 package for more complex graphics. This is designed for the advanced users only and not recommended.

## 3. Results

We developed the* Guitar* R/Bioconductor package for gene annotation guided transcriptomic analysis of RNA-related genomic features. It is currently and publicly available from Bioconductor. In this section, we show the application of* Guitar* package to the analysis of RNA methylation sites denoted as genomic features with a few examples. More information is available from the documentation of the* Guitar* R/Bioconductor package.

### 3.1. Case Study 1: RNA N6-Methyladenosine from MeRIP-Seq

In the first example, we study MeRIP-Seq data [10] profiling of the transcriptome RNA N6-methyladenosine (m^6^A) [18, 19] sites in human HepG2 cell lines. The RNA m^6^A methylation sites are obtained with exomePeak R/Bioconductor package [12]. Compared with other peak calling algorithms, one added flexibility of the exomePeak package is to detect only the highly methylated sites. With a larger “IP/input ratio,” exomePeak will report only the highly methylated sites; that is, a higher proportion of a specific kind of RNA molecule carries methylation at this site.

**Figure 3:**
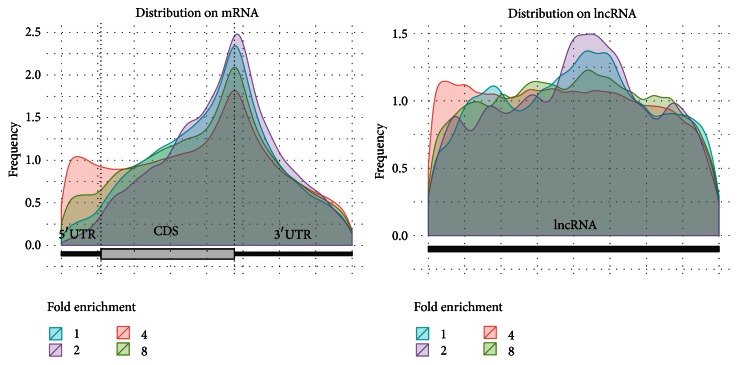
m^6^A sites on mRNA and lncRNA. In mRNA, the strongest binding sites (“IP/input ratio” larger than 8) are highly enriched near stop codon side of 3′UTR and deficient on TSS (transcription starting site) side of 5′UTR and the phenomena are more prominent than lowly methylated sites. In contrast, the m^6^A sites are almost uniformly distributed on lncRNA despite the “IP/input ratio” specified. Please note that, in this figure, the size of 5′UTR, CDS, and 3′UTR reflects their true width within the transcriptome, so the 5′UTR region is much shorter compared with the other two components. This result is based on peaks called on human HepG2 dataset [10].

We implemented exomePeak setting different “IP/input ratio” (1, 2, 4, and 8), and we then examined the distributions of the peaks in different exonic regions at each enrichment threshold. As shown in Figure 3, the detected m^6^A sites with more than 8 times of enrichment are overly present near the stop codon but highly deficient near transcription starting site (TSS) and on 5′UTR. The different distribution patterns of the highly and weakly methylated sites may indicate function versatility and call for more specialized analysis targeting the sites with different methylation level. In contrast to distribution preference of m^6^A sites on mRNA, it is almost uniformly (or randomly) distributed on lncRNA regardless of the “IP/input ratio” specified.

In the aforementioned example, we implemented the default.

### 3.2. Case Study 2: RNA 5-Methylcytosine from BS-Seq

In previous study, we confirmed that RNA m^6^A methylation sites are enriched near stop codon and discovered that highly enriched m^6^A methylation sites are depleted on 5′UTR. Next, we study 5-methylcytosine (m^5^C) [20, 21] profiled with RNA BS-Seq experiment [11, 22]. Different from the relatively well-studied DNA m^5^C methylation [23, 24], the functions of RNA m^5^C on mRNA are still largely elusive [20], and its global distributions on mRNA and lncRNA are poorly characterized so far.

The bisulfite sequencing data characterizing the RNA m^5^C methylation profiles in mouse embryo fibroblast [25] was directly obtained from GEO (GSE44359) and then processed with trim_galore [26] to remove low-quality reads and adaptor sequences; the processed reads are then aligned to mouse mm10 genome assembly with Bismark [27] and 424627 Cytosine (C) residuals are called with at least 5 reads aligned. To analyze the distribution of methylated C residuals, we further divided all the reported C residuals into 3 groups based on their methylation level with a binomial test using the average methylation probability (1.65%) of the entire transcriptome at confidence level 0.05.

**Figure 4:**
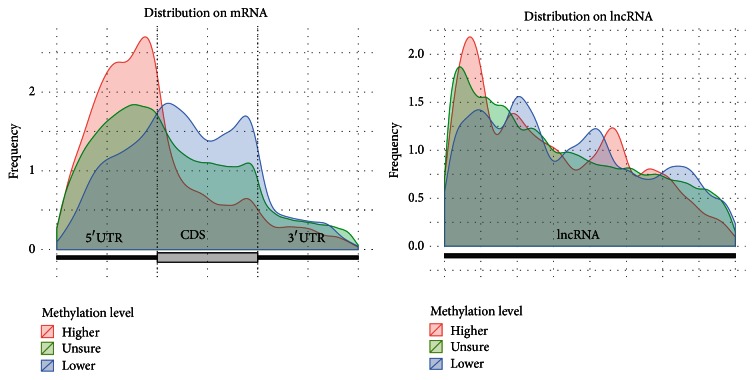
Distribution of RNA m^5^C residuals on mRNA and lncRNA. We divided all the Bismark reported C residuals into 3 groups; that is, 9514 residuals with methylation level higher than 1.65%, 8600 lower, and 406513 residuals cannot be determined with statistical significance. We can see that, for all 3 groups, there exists a strong 5′ bias on the mouse RNA BS-Seq data on both mRNA and lncRNA. The highly m^5^C methylated residuals are strongly enriched at 5′UTR of mRNA.

It can be seen from Figure 4 that the distribution of m^5^C on both lncRNA and mRNA has a very strong 5′ bias; that is, more reported C residuals from BS-Seq data are on the 5′UTR region. The distribution patterns of 3 groups of residuals with different methylation level are quite different on mRNA, with highly methylated Cs (higher than 1.65% with 0.05 confidence level) prominently enriched on 5′UTR and near start codon. A cytosine residual located on 5′UTR is around 1 times more likely to be methylated than that on CDS and 0.68 times more likely than that on 3′UTR. While previous study indicates RNA m^5^C is enriched on both 5′UTR and 3′UTR, our analysis with increased resolution indicates the enrichment is a lot stronger on 5′UTR than 3′UTR [20]. We also notice the pattern is complementary to the distribution of m^6^A, which is enriched near stop codon and 3′UTR [8] (see Figure 3). These observations suggest that RNA m^5^C and m^6^A may work in a complementary manner. On lncRNA, however, despite the overall 5′ bias, there is no apparent difference in their distribution patterns revealed.

**Figure 5:**
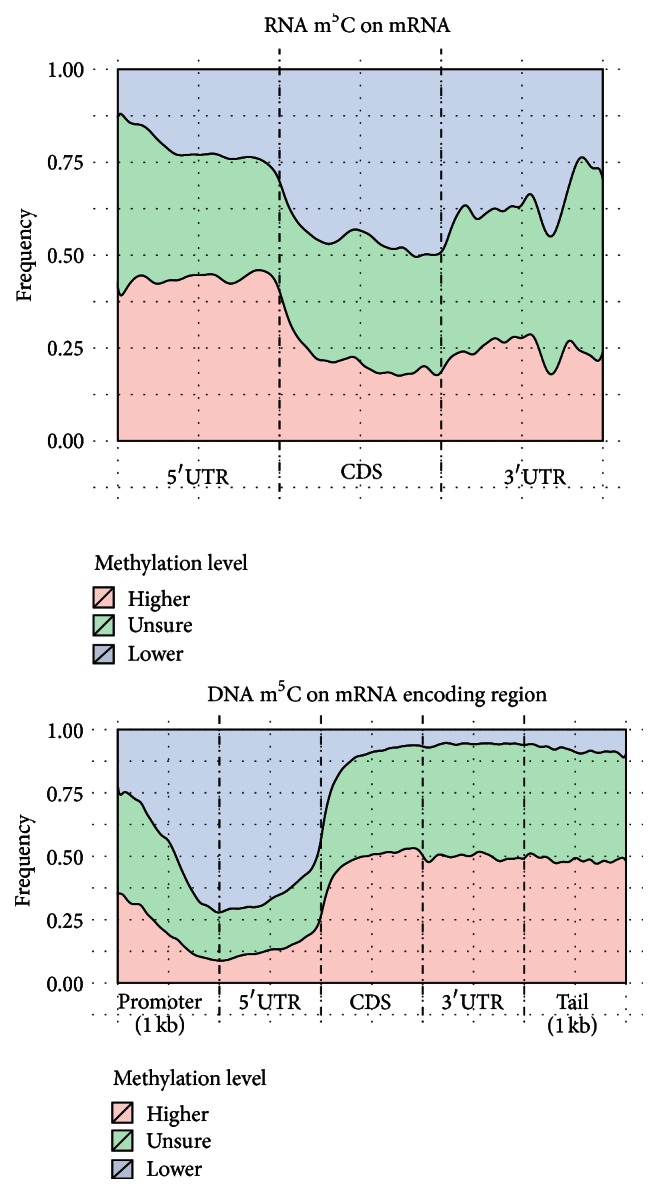
Distribution of DNA and RNA m^5^C residuals. While C residuals of mRNA are likely to be methylated on 5′UTR, it is opposite on DNA. On DNA, the start codon and 5′UTR regions are less likely to be methylated compared with other regions to allow the initiation of transcription process.

We in the following compared the relative distribution of highly and lowly m^5^C methylated C residuals on RNA and DNA. The same processing pipe line as described previously is applied to mouse embryo fibroblast whole genome bisulfite sequencing data [25], which profiles the DNA m^5^C methylation rather than that on RNA. A total of 3641243 cytosine residuals are reported by Bismark, and they are then divided into 3 groups (988335, 1966017, and 686891 residuals, resp.) based on whether their methylation level is significantly higher or lower than the average reported DNA methylation level (65.79%) with a binomial test at 0.05 confidence level. The distributions of the 3 groups of cytosine residuals are still profiled by* Guitar* package with neighborhood DNA regions included, and the results are shown in Figure 5. We can see that, compared with RNA methylation profile, where the highly methylated residuals are enriched on 5′UTR, the DNA methylation profile shows a clear oppose pattern, with the lowly methylated residuals enriched on 5′UTR and peaked near the start codon to enable transcription initiation. This observation indicates DNA and RNA methyltransferase complexes probably have quite different sequence specificity, even though some key enzyme genes, such as Dnmt2 [28, 29], are shared between them [30].

## 4. Discussion and Conclusion

Currently, most biological features are represented with genome-based coordinates, making it rather tedious for comparing with transcriptomic landmarks. We developed a* Guitar* R package, which can be a useful tool for analyzing RNA-related genomic features, especially RNA methylation. Built upon the highly efficient* GRangesList* structure and* GenomicFeatures* R/Bioconductor packages,* Guitar* can efficiently process millions of genomic features within minutes for efficient transcriptomic analysis. It may automatically download gene information from UCSC genome browser, including neighborhood DNA regions, and allocate the weight of ambiguous features. The developed* Guitar* coordinates also provide low level conversion from transcript-based coordinates to genome-based coordinates, which should facilitate various customized analysis. Nevertheless, there are still a number of issues remaining to be addressed.

Firstly, highly abundant transcripts and ambiguous genomic features may dominate the result from* Guitar* package. Currently, all genomic features and all RNA transcripts have the same weight; this is fine when the genomic features are biological features such as RNA methylation sites, but it may cause problem when the genomic features are NGS mapped reads, such as RNAseq reads, because some genes are a lot more highly expressed than the other genes, and the reads generated from these genes may dominate the result. A more robust approach may be to use the median abundance of all RNA transcripts rather the summed density. It is also possible to develop a boxplot-like visualization scheme in the future to indicate the estimated higher and lower bounds for the distribution of tested features.

Secondly, the developed normalization scheme within* Guitar* package only facilitates the comparison of feature distribution on mRNA transcripts or on lncRNA transcripts. A cross-comparison between mRNA and lncRNA is not supported so far. In general, it is still very difficult to rigorously contrast the distribution of genomic features on mRNA and lncRNA due to their intrinsic difference. Compared with mRNA, lncRNA usually has lower expression level and less number of exons and is less conserved in sequence.

Thirdly, because of the heterogeneity of transcriptome and the discrepancy between transcriptomic and genomic features, ambiguity arises and cannot be resolved perfectly. Previous study of transcriptomic distribution of RNA methylation relies on the longest transcript to eliminate ambiguity in feature assignment, which may require further justification on why the longest transcript should be the canonical one. A more reasonable solution may be to rely on the most abundant isoform transcript; however, the isoform quantification is usually a difficult problem, and such information may not be handy and sometimes may be unavailable at all. Currently,* Guitar* package adapts two strategies to address this issue. Firstly, highly ambiguous genomic features or transcripts are excluded from the analysis. Secondly, the weight of ambiguous mapping is evenly divided among transcripts. It should be possible to develop a more rigorous formulation, for example, with fuzzy system model [31–33], to further improve the allocation of weight from ambiguous overlapping.

Fourthly, during the development process of the* Guitar* package, we also realized the* Guitar* coordinates may potentially be used for accessing the data quality of MeRIP-Seq datasets. The reproducibility, uniformity, homogeneity, and the information entropy within RNA-related sequencing data (RNA-Seq, MeRIP-Seq, and BS-Seq) can be conveniently assessed with the help of* Guitar* coordinates; however, this application has not been explored so far and will be our future work.

Despite the aforementioned limitations, the* Guitar* package is capable of sketching the approximate transcriptomic distribution of millions of genomic features within minutes in a single line of command. We believe that the newly developed* Guitar* coordinates and the* Guitar* package have achieved stable performance on the data tested and should effectively facilitate the analysis of RNA methylation data and other RNA-related biological features.

## Supplementary Material

Supplementary Material contains information of Figure S1 and Table S1, in which we Compare different settings of ambiguity filter.

## Additional Points

Project name is* Guitar.* Project homepage is http://bioconductor.org/packages/Guitar/. Operating system(s) is Platform Independent. Programming language is R. License is GPL-2. For manual and guide, please see supplementary materials.
