# Supplementary material for: Guitar: An R/Bioconductor Package for Gene Annotation Guided Transcriptomic Analysis of RNA-Related Genomic Features
Source: Biomed Res Int. 2016 Apr 28;2016:8367534. doi: 10.1155/2016/8367534 (PMC4864564; doi:10.1155/2016/8367534)
Supplement: Supplementary file 1 — Supplementary Material contains information of Figure S1 and Table S1, in which we Compare different settings of ambiguity filter. [file 8367534.f1.pdf]

Supplementary Material

for Guitar: an R/Bioconductor package for gene annotation guided transcriptomic analysis of RNA related genomic features

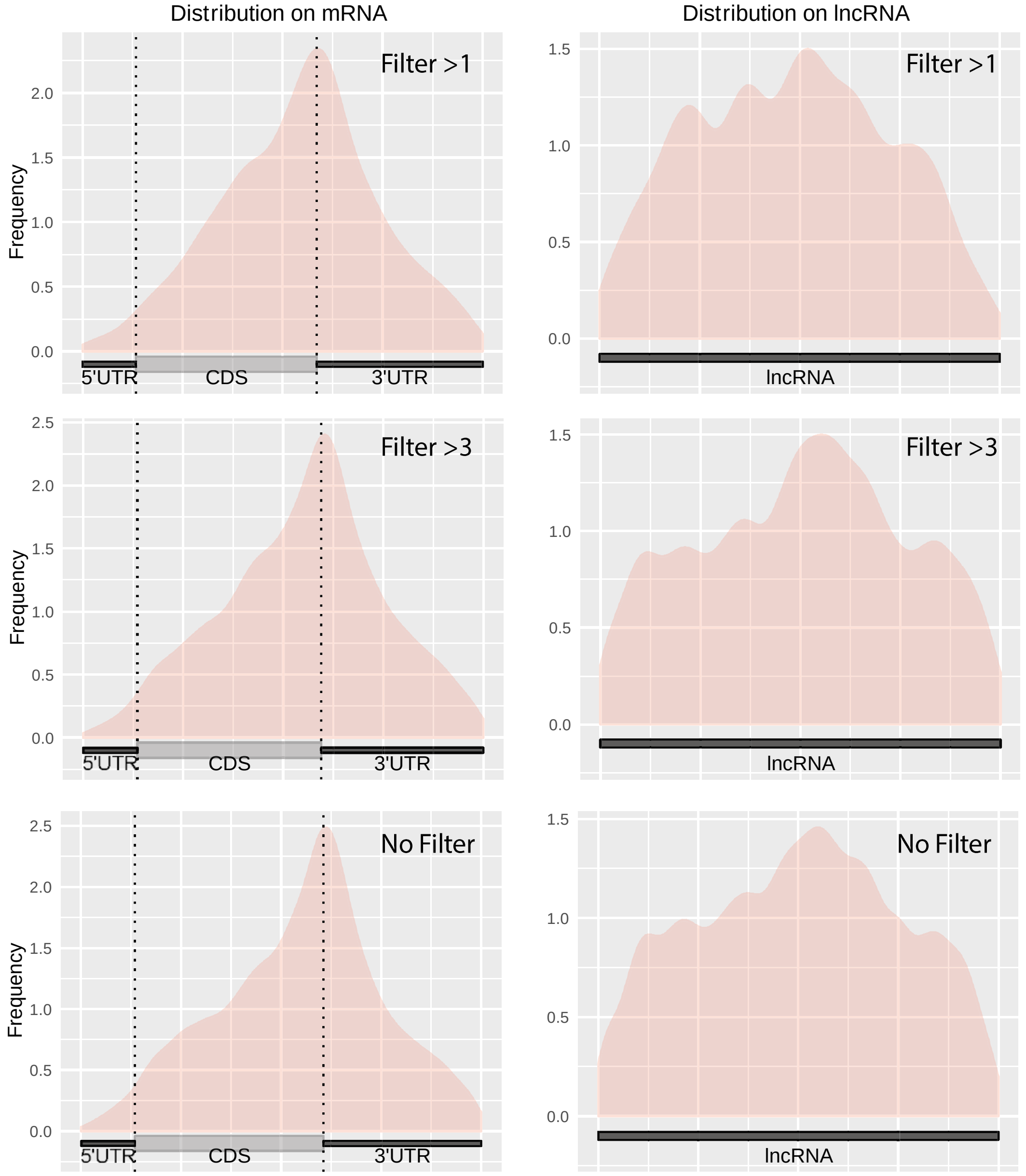

Figure S1. Comparing different setting of ambiguity filter. We still apply Guitar package to the RNA m^6A sites shown in case study 1 with different settings on the ambiguity filter. As we can see in the above figure, the impact of different settings is minimal; however, as shown in Table S1, the computation time and memory usage can be quite different. On one hand, more stringent ambiguity filter retain less number of transcripts and thus requires less computation resources; on the other hand, transcripts with high degree of ambiguity can serve as both the source of information or noise. It is important to adjust this parameter based on the actual data used and the problem of study.

Table S1 Different Setting of Ambiguity Filter

**Ambiguity Filtered**	**Run Time**	**Memory Usage (MB)**	**# of Transcripts Used (mRNA + lncRNA)**
Unlimited (no filter)	167.52	320.9	42337 + 12678
>3 (default)	91.12	162.2	20302 + 8184
>1 (extreme)	51.82	69.4	7706 + 5090

Note: Guitar package applied to RNA m^6A sites from case study 1 with different settings on the ambiguity filter.
